# Solvents of Gall-Stones

**Published:** 1880-01

**Authors:** 


### SELECTIONS.

---

### SOLVENTS OF GALL-STONES.

IN the *Boston Medical and Surgical Journal* for Oct. 23, 1879, Dr. T. H. Buckler directs attention to the recent triumphs of and accessions to surgery with reference to the recent boasts that already the gall-bladder had been invaded by the surgeon's knife, with the object of removing therefrom biliary calculi. Dr. Thomas, in the able address delivered before the Gynecological Society, related this as an example to illustrate the progress and paramount importance of surgery. Dr. Buckler, however, affirms that if there is any one thing that does and must forever belong exclusively to the department of practical medicine, it is the ready means physicians have at command of being able always to dissolve in the gall-bladder cholesteric gall-stones with as much certainty as if these same calculi were in a glass tumbler.

Eight or ten years ago a paper was published in *Ray's Journal* recommending chloroform in doses of from five to sixty drops every four to six hours, as a sure means of dissolving calculi in the gall-bladder, however large and numerous they might be.

In the *American Journal of Medical Sciences* for July, 1867, Dr. Buckler advised the use of the succinate of iron as a solvent of gall-stones and of cholesteric fat, whether in the arteries or elsewhere. This preparation contains more nascent appropriable oxygen than any other known therapeutic agent, and of all the feruginous articles is one of the very best for malarious cachexy, or for any other condition where the blood-globules diminish or need rehabilitation. In all hepatic diseases in which nitric and hydrochloric acids are usually prescribed, the succinate of iron will be found more efficacious.

In critical and urgent cases of gall-stone, where no time can with safety be lost, Dr. Buckler prefers the conjoint use of terchloride of formyl, and Stewart's preparation of the succinate of iron. In the last three cases treated successfully, the administration of both chloroform and succinate of iron was commenced as soon as the existence of a gall-stone was established beyond a reasonable doubt, giving the former in doses of ten drops every four hours, and of the latter a teaspoonful half an hour after each meal. In two instances the patients were able to take a teaspoonful of chloroform every six hours without any ill effect. These doses dissolved the calculus within the space of a single week.

Dr. Buckler states that he has seen a number of cases of gallstone which were successfully treated with chloroform to dissolve the cholesterine existing in the gall-bladder at the time, and causing paroxysms of pain amounting to positive anguish.

After existing calculi have been dissolved, then, to overcome the cholesteric diathesis, and to prevent the formation of other stones, the patients were kept on teaspoonful doses, thrice daily, of succinate of iron for a period of four or six months.

Of all the certainties of medicine, there is nothing more absolutely sure than that chloroform will in every instance dissolve calculi in the gall-bladder. When taken into the stomach it passes with the blood of the portal circulation, out of which bile is made, directly to the acini of the liver, and is carried with the newly-found biliary fluid to the gall-bladder, where its solvent power is effectual. Sometimes chloresterine clogs the acini and lesser biliary ducts, producing jaundice, in which cases this otherwise obstinate trouble is promptly relieved by giving chloroform. Ether has been recommended for the same purpose, but is not as reliable, owing to the difference in the specific gravity of these two agents, ether being the most diffusible, and floating in water, while chloroform sinks in it. According to Dr. Buckler's observation, chole-lithiasis is found four times in women where it occurs once in the opposite sex.

The surgeon has a fair field for trial of the proposed solvents before resorting to an expedient so hopelessly perilous as the knife.

[We have during the last eight years treated with complete success more than twenty cases of chole-lithiasis by the use of succinate of the per-oxide of iron alone.—Eds.]

